# Modulation of Estrogen Receptor Alpha (ERα) and Tumor Suppressor Gene BRCA1 in Breast Cancer Cells by Bazedoxifene Acetate (BZA)

**DOI:** 10.3390/cancers16040699

**Published:** 2024-02-07

**Authors:** Monica Szmyd, Aisha Zanib, Victoria Behlow, Erin Hallman, Samantha Pfiffner, Raquel Yaldo, Nina Prudhomme, Katelyn Farrar, Sumi Dinda

**Affiliations:** 1 Department of Clinical and Diagnostic Sciences, School of Health Sciences, Oakland University, Rochester, MI 48309, USA; monica.szmyd@corewellhealth.org (M.S.); azanib@oakland.edu (A.Z.); victoria.behlow@corewellhealth.org (V.B.); hallmane@msu.edu (E.H.); samantha.pfiffner@med.wayne.edu (S.P.); raquelyaldo@oakland.edu (R.Y.); kfarrar@oakland.edu (K.F.); 2 Department of Foundational Medical Sciences, Oakland University William Beaumont School of Medicine, Rochester, MI 48309, USA; 3 Institute of Stem Cell and Regenerative Medicine, Oakland University, Rochester, MI 48309, USA; 4 Center for Biomedical Research, Oakland University, Rochester, MI 48309, USA

**Keywords:** ERα, BRCA1, estrogen receptor, tumor suppressor gene, bazedoxifene acetate, breast cancer, selective estrogen receptor modulators

## Abstract

**Simple Summary:**

This study focused on exploring the biochemical attributes of bazedoxifene acetate (BZA), a promising candidate for breast cancer treatment. It functions as an osteoprotective agent by maintaining bone density while concurrently displaying antiestrogenic properties in breast tissue—a valuable characteristic for breast cancer endocrine therapy. Our study investigated how BZA affects certain key proteins associated with Estrogen Receptor Alpha (ERα) and tumor suppressor gene BRCA1 in breast cancer cells. The findings demonstrated a substantial reduction in the levels of these proteins, signifying the potential therapeutic relevance of BZA in managing breast cancer. Furthermore, BZA exhibited antiproliferative properties, suggestive of its capacity to inhibit cell growth. Understanding the role of BZA in regulating these proteins is crucial for advancing breast cancer treatment and hormone receptor research.

**Abstract:**

Selective estrogen receptor modulators (SERMs) are steroid analogs with dual functionality, acting as partial estrogen receptor agonists to preserve postmenopausal bone density and as estrogen receptor antagonists in breast tissue. Bazedoxifene acetate (BZA) is an FDA-approved, third-generation SERM used in the treatment of osteoporosis in women. It demonstrates potential as a therapeutic option for breast cancer patients undergoing endocrine therapy. Our study aimed to assess BZA’s effects on Estrogen Receptor Alpha (ERα) and tumor suppressor gene BRCA1 in T-47D and MCF-7 breast cancer cells, using Western blots, cellular viability, apoptosis assays, and RT-qPCR. Cells were cultured in 5% charcoal-stripped fetal bovine serum for six days to deplete endogenous steroids. Following a 24 h exposure to 2 µM BZA (optimal concentration determined from 1 nM–2 µM studies), Western blot analyses revealed reduced ERα and BRCA1 protein levels in both cell lines. ERα decreased by 48–63% and BRCA1 by 61–64%, indicating sensitivity to antiestrogens. Cytolocalization of ERα and BRCA1 remained unchanged after BZA and 17-β-estradiol (E_2_) treatment. ESR1 mRNA expression correlated with Western blot findings. Image cytometric analysis using the stain, propidium iodide, detected decreased cellular proliferation in T-47D and MCF-7 cells following a 6-day treatment ranging from 1 nM to 2 µM BZA. BZA treatment alone led to a tenfold reduction in cellular proliferation compared to estrogen-treated cells, suggesting antiproliferative effects. Understanding BZA’s modulation of BRCA1 and ERα, along with their mechanistic interactions, is vital for comprehending its impact on breast cancer tumor suppressors and hormone receptors.

## 1. Introduction

Breast cancer, primarily impacting women, is recognized as the most frequently diagnosed malignancy worldwide. It has surpassed lung cancer to become the leading global cancer diagnosis, accounting for one in eight of all cancer cases [1]. Estrogens are widely recognized for their role in promoting the proliferation of neoplastic breast epithelium [2]. Breast cancers may display hormone dependency, with Estrogen Receptor Alpha (ERα) acting as the primary receptor for 17-β-estradiol (E_2_), the predominant form of estrogen produced largely in the ovaries of women during their reproductive years. ERα is present in no more than 10% of normal breast epithelium; however, in breast cancer tissue, it is present approximately 50–80% of the time [3,4]. Studies have demonstrated that aberrant ERα expression contributes to both the development and progression of hormone-dependent breast cancer [5,6]. Upon hormonal stimulation, these receptors translocate into the cell nucleus, where they interact with DNA to either express or repress genes involved in fundamental cellular processes [7].

The function of ER in breast cancer is well-established, primarily promoting proliferation and cell survival. However, its involvement in metastasis is less established, which highlights the importance of continued research efforts. ER’s contribution to metastasis is mainly characterized by the loss of its classical function, acquisition of crosstalk with other signaling pathways, or alterations in ER coregulators [8]. 

In the presence of ERα, the mitotic activity of breast cancer cells is drastically increased in patients with mutated tumor suppressor genes [3,9]. The tumor suppressor, Breast Cancer Gene 1 (BRCA1), is responsible for DNA double-strand break repair through homologous recombination, which protects cells from harmful mutations that could otherwise lead to the initiation and progression of breast cancer [10,11]. In addition, studies have shown BRCA1 to antagonize ERα, which provides further protective effects in cancerous breast epithelium. In fact, BRCA1 has been shown to be a major antagonizing factor in the pathway between estrogen and estrogen receptor signaling [12]. Mutations in the BRCA1 gene impair DNA repair, increasing susceptibility to various cancers, especially breast cancer [6]. Notably, a mutation in the BRCA1 gene is associated with a 60–80% likelihood of developing breast cancer in women [13]. In both the T-47D and MCF-7 cell lines, the BRCA1 gene is found in the wild-type form. Due to the intricate interactions between ERα and BRCA1, it is essential to study the effect of these genes and their corresponding proteins, particularly when BRCA1 is mutated in breast cancer cells. This type of investigation can provide a deeper understanding of the molecular pathway of action and therapeutic potential of bazedoxifene acetate (BZA) [14,15].

In postmenopausal women, selective estrogen receptor modulators (SERMs) have a dual mechanism of action. They can function as partial estrogen receptor agonists and demonstrate osteoprotective effects while also exhibiting antiestrogenic effects in breast tissue [16]. The use of SERMs in the prevention or treatment of osteoporosis does not adversely impact breast cancer risk and may confer protective benefits [17]. 

Tamoxifen (TAM) is a SERM that has been used in the treatment of breast cancer for over 40 years. It has been used as the endocrine treatment of choice for women with ER-positive breast cancer, irrespective of stage, for several decades due to its excellent efficacy and relatively mild side effect profile [18]. Moreover, studies have depicted a 38% decrease in breast cancer incidence in women treated with tamoxifen versus placebo, regardless of age [19,20]. Due to its action as a SERM, TAM exhibits both estrogenic and anti-estrogenic effects, which are dependent upon the tissue type at the site of action. Moreover, in breast tissue, TAM exhibits anti-estrogenic effects by binding to ERα and inhibiting cellular proliferation [21]. While TAM proves beneficial for breast cancer treatment, its prolonged use presents a significant limitation due to an increased risk of endometrial cancer [22,23]. This highlights the need to study newer generation SERMs and treatment options for breast cancer.

Raloxifene (RAL), also a SERM, is used as a treatment in various disease states but is notable for reducing the progression of breast cancer [24]. RAL acts in preventing the proliferation of breast cancer by exhibiting anti-estrogenic effects, similarly to TAM, by binding to ERα and therefore preventing estrogen from binding to that site [25]. Given the clinical usage, demonstrated effectiveness, and our previous work in our lab with both TAM and RAL, the inclusion of these compounds is helpful for facilitating a meaningful interpretation and comparison of the effects of BZA [26,27]. In addition to TAM and RAL, other newer SERM options include ospemifene, bazedoxifene, arzoxifene, and lasofoxifene [16]. The newer options have the potential for increased potency and efficacy when compared to previous SERMs, with studies currently underway to determine their impact clinically. 

Bazedoxifene acetate (BZA), a third-generation SERM, has FDA approval to treat osteoporosis when in combination with conjugated estrogens. However, it lacks approval for use as a treatment option for breast cancer. BZA has unique structural characteristics with respect to TAM, which is used in breast cancer therapy, and RAL, which is utilized for osteoporosis treatment [28]. BZA demonstrates selective activity related to the skeletal system; however, a SERM with sustained antagonistic activity on breast cancer tissue has yet to be developed. In vitro studies of the MCF-7 cell line have shown that BZA inhibits breast cancer cell proliferation in a dose-dependent manner when cells were co-treated with BZA and E_2_; however, when cells were treated with BZA alone, no significant effect was observed [29]. This observation leads to the inquiry into the mechanism behind BZA’s interaction with the estrogen–estrogen receptor pathway in breast cancer epithelium. Currently, BZA is being utilized in clinical trials that aim to prevent breast cancer in peri- and postmenopausal women, with a proposed mechanism of enhancing apoptosis within breast epithelium [30,31]. Studies have shown that estrogen-induced cell growth and proliferation are antagonized by BZA through inhibition of gene expression [17]. Although this interaction was previously thought to be through competitive inhibition, a study using microarray analysis demonstrated a formation of heterodimers between BZA and estrogen, which together control cellular transcription [32].

Additional investigations are necessary to comprehensively assess the impact of BZA on breast cancer cells. Therefore, determining the effects of BZA on steroid receptors and tumor suppressor genes is essential. This study was designed to evaluate the influence of BZA on ERα and BRCA1 in T-47D and MCF-7 breast cancer cell lines, utilizing Western blot analyses, cellular viability assessments, apoptosis assays, and Reverse-Transcription Quantitative Real-Time Polymerase Chain Reaction (RT-qPCR) studies.

## 2. Materials and Methods

### 2.1. Cell Culture and Treatment with Ligands

The human breast cancer cell lines, T-47D and MCF-7, were obtained from the American Type Culture Collection (ATCC; Manassas, VA, USA). The cells were routinely cultured and incubated at 37 °C with 5% carbon dioxide (CO_2_). T-47D cells were treated with RPMI-1640 medium (HyClone; Logan, UT, USA), supplemented with 10% fetal bovine serum (FBS) (HyClone; Logan, UT, USA). MCF-7 cells were cultured in Eagle’s Minimum Essential Medium (Corning; Corning, NY, USA), also supplemented with 10% FBS. The culture media, enriched with growth factors and exogenous steroids to promote cell growth and proliferation, underwent regular replacement every 48 h for both cell lines. Once the cells were confluent, the cells were subcultured into 6-well plates (200,000 T-47D cells per well and 200,000 MCF-7 cells per well), and the media was replaced with 5% dextran-coated charcoal-stripped FBS (DCC-FBS; Santa Cruz Biotechnology.; Dallas, TX, USA). DCC-FBS-stripped serum depletes the cells of any exposure to endogenous steroids and growth factors. This ensured that the cells maintained their basal metabolic rate throughout the treatment with the BZA compound (courtesy of Pfizer Inc.; New York, NY, USA), establishing that the observed effects on the cells were attributable to the compound itself and not other factors in the culture media. The cells were cultured for six days and then exposed to 2 μL of ligands for 24 h. To determine the optimal concentration of the compound, a concentration study was conducted for each cell line. In this study, the cells were treated with 2 μL of varying concentrations of the BZA compound (1 nM to 2 μM). Subsequently, a hormone study was performed for each cell line, where 2 μL of hormones and antihormones were administered in combination with the optimal concentration of the compound, which was determined to be 2 μM BZA for both MCF-7 and T-47D cell lines (based on the aforementioned concentration studies that were performed). 

### 2.2. Protein Extraction and Quantification

After a 24 h treatment period, cellular proteins were extracted. The 5% stripped serum was aspirated and followed by a wash using Hank’s Balanced Salt Solution (HBSS; HyClone; Logan, UT, USA), which was then aspirated. Subsequently, the cells were then lysed using an extraction buffer to maintain protein stability. Protein extraction utilized a lysis buffer known as radioimmunoprecipitation assay buffer (RIPA), phenylmethanesulfonyl fluoride (PMSF), and a protease inhibitor cocktail (Santa Cruz Biotechnology, Inc.; Dallas, TX, USA). Following the addition of the buffer, the lysates were subjected to high-speed centrifugation at 15,000 RPM for a duration of 15 min at a temperature of 4 °C. The supernatant of each sample was then isolated and subjected to the Bradford protein assay method (Bio-Rad; Hercules, CA, USA). The protein concentration of each sample was determined utilizing a spectrophotometer, and the extracted protein samples were standardized to ensure equal amounts of protein content for subsequent gel electrophoresis and other downstream analyses.

### 2.3. SDS-PAGE and Western Blot Analyses

The extracted proteins were subsequently separated according to molecular weight by running sodium dodecyl sulfate–polyacrylamide gel electrophoresis (SDS-PAGE), enabling the isolation of the target proteins for Western blot analyses. This technique began with the denaturation of proteins in the supernatant to their primary structure. The supernatant was heated to 85 °C for three minutes. Each sample was then loaded into a 7.5% polyacrylamide gel at consistent concentrations, as determined by the previously mentioned protein assay. A method known as electro-blotting allowed the proteins in the gel to be transferred to an Immobilon polyvinylidene fluoride (PVDF) membrane (Millipore; Bedford, MA, USA). Subsequently, the membrane underwent a 30 min wash in tris buffered saline (TBS-Tween 0.1%) and was then blocked with 5% nonfat dry milk for one hour. This blocking effectively prevented non-specific proteins from binding to the membrane. To detect ERα and BRCA1, primary antibodies were used: anti-mouse monoclonal antibody (Santa Cruz Biotechnology, Inc.; Dallas, TX, USA) and anti-rabbit polyclonal (Cell Signaling; Danvers, MA, USA). This was followed by three 10 min washes using TBS-Tween and a subsequent 30 min re-blocking step with 5% nonfat dry milk. In order to distinguish the primary antibodies, a secondary goat anti-mouse IgG2a antibody and a secondary anti-rabbit antibody (Jackson Laboratories; Bar Harbor, ME, USA) were used, respectively. The specific bands for ERα and BRCA1 were visualized using the enhanced chemiluminescence (ECL) technique (Advansta; Menlo Park, CA, USA). The protein bands were then viewed using the ChemiDoc XRS+ molecular imaging system (Bio-Rad; Hercules, CA, USA). After immunoblotting, the PVDF membranes were stained with coomassie blue to confirm the accurate normalization of total protein levels and to validate the complete transfer of proteins. The protein band density from the Western blots was quantified using the Image Studio Lite program version 3.1 (LI-COR Biosciences; Lincoln, NE, USA).

### 2.4. Reverse Transcription Quantitative Real-Time PCR (RT-qPCR)

Utilizing the TRIzol reagent (Invitrogen; Carlsbad, CA, USA), total ribonucleic acid (RNA) was extracted from T-47D cells, following the manufacturer’s guidelines. The resulting genomic deoxyribonucleic acid (gDNA)-free total RNA was subjected to reverse transcription, generating copy deoxyribonucleic acid (cDNA). This conversion was executed using the iScript cDNA Synthesis Kit (Bio-Rad Laboratories; Hercules, CA, USA) in compliance with the manufacturer’s instructions. Prior to the RT-qPCR analysis of the reverse-transcribed cDNA, a tenfold dilution of the cDNA was prepared using PCR-grade water. Subsequently, RT-qPCR was conducted in a 96-well plate, utilizing the Bio-Rad CFX384 Real-Time PCR Detection System. The assay incorporated numerous controls, such as a no template control (NTC), a reverse transcription negative (RT-), and a no reverse transcription (NRT). These controls were implemented to identify potential reagent contamination and the presence of gDNA. Data analysis involved the normalization of specific gene expression relative to an endogenous control, achieved through the utilization of the ΔΔCq method. This process entailed the comparison of the quantification cycle (Cq) value of the target gene with that of an endogenous control, a reference gene exhibiting stable expression levels. Expression of the Actin Beta (ACTB) genes served as the chosen endogenous controls. The ΔΔCq method provided a precise means for quantification and assessment of alterations in gene expression levels under different experimental conditions. The normalized ΔCq values from treated samples were compared to the reference stripped control (Cs) to derive ΔΔCq values, which were then used to calculate the relative fold change as compared to the control. The quantification of Estrogen Receptor Alpha Gene (ESR1) and BRCA1 mRNA levels was carried out using RT-qPCR. Both T-47D and MCF-7 cell lines were subjected to a 24 h treatment regimen with or without 2 µM of BZA, E_2_, RAL, and ICI. The presented data represent the mean ± standard error of mean (SEM) derived from a minimum of three distinct experiments, with each experiment involving three replicates.

### 2.5. Cell Proliferation Assays

Following treatment with ligands of varying concentrations, cell viability assays were employed to quantify the population of viable cells. Each assay was performed a total of three times for both MCF-7 and T-47D cell lines. The studies were conducted in 12-well culture plates, each initially seeded with 3.0 × 10^4^ cells per well. The cells were nurtured in 1 mL of media enriched with 10% FBS for the initial two days. During the following six days, the growth factor-enriched media was replaced with stripped serum media, and the cells were subjected to ligand treatments over two-day intervals. This experimental design allowed for an evaluation of how different ligand concentrations influenced the overall viability of the cells throughout the course of the study. Ligand treatments composed of 1 nM to 2 µM BZA were implemented with various hormones and antihormones. Following trypsinization, the cells were carefully extracted from their respective culture wells. To allow for fluorescence-based analysis, they were then stained with propidium iodide (PI; Sigma Aldrich; St. Louis, MO). Subsequently, the stained cells underwent detailed image cytometry analysis using the Cellometer Vision CBA Image Cytometry System software version 216 (Nexcelom Bioscience LLC.; Lawrence, MA, USA). The number of dead cells found in comparison to total cells was quantified and used to calculate cell viability.

### 2.6. Apoptosis Assay

Growth studies with cell proliferation assays were performed as previously discussed. Each assay was conducted three times for each cell line. Following proper cell culture and preparation, the cells were treated with PI and fluorescein isothiocyanate-conjugated Annexin V dyes (BioLegend, San Diego, CA, USA). PI was used to discern dead cells, whereas Annexin V identified cells in the early stages of apoptosis. Subsequently, the stained cells were subjected to analysis through imaging cytometry. This analysis was carried out using the Cellometer Vision CBA Image Cytometry System software version 216 (Nexcelom Bioscience LLC.; Lawrence, MA, USA). The software’s fluorescence threshold was set at 0% in order to assess the overall fluorescence emitted by each counted cell in the captured images. The recorded fluorescence intensities were exported to Microsoft Excel and converted into an FCS file format. FCS Express-5 Flow Research Edition was employed for the comprehensive analysis and interpretation of the fluorescence data (De Novo Software; Pasadena, CA, USA).

### 2.7. Immunofluorescence and Confocal Microscopy 

T-47D and MCF-7 cells were plated directly onto coverslips in 12-well plates and were subjected to immunolabeling to visualize the ERα within the cells. The spatial arrangement of the fluorescent structures was assessed using a Nikon Digital Eclipse C1-Plus confocal microscope (Nikon Instruments; Melville, NY, USA). To further refine the obtained images, NIS Elements AR software version 4.60 was used to facilitate noise reduction and reconstruction of three-dimensional images (Nikon Instruments; Melville, NY, USA). 

### 2.8. Statistical Analyses

The results from Western blot analyses, RT-qPCR, and cell viability assays were expressed as a mean ± standard error of the mean (SEM). Statistical significance was determined by the Kruskal–Wallis test and followed by post hoc analysis using the Mann–Whitney U-Test. Differences were considered significant at * *p* < 0.05, ** *p* < 0.01, and *** *p* < 0.001. Statistical analyses were carried out using SPSS for Windows version 11.5 (SPSS Inc.; Chicago, IL, USA).

## 3. Results

### 3.1. Concentration-Dependent Effects of BZA on ERα and BRCA1 Levels

**Figure 1:**
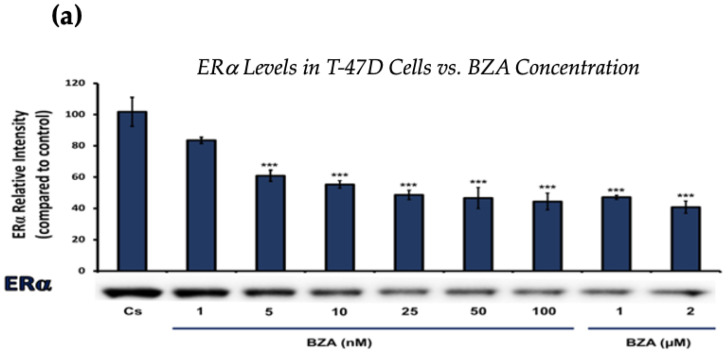

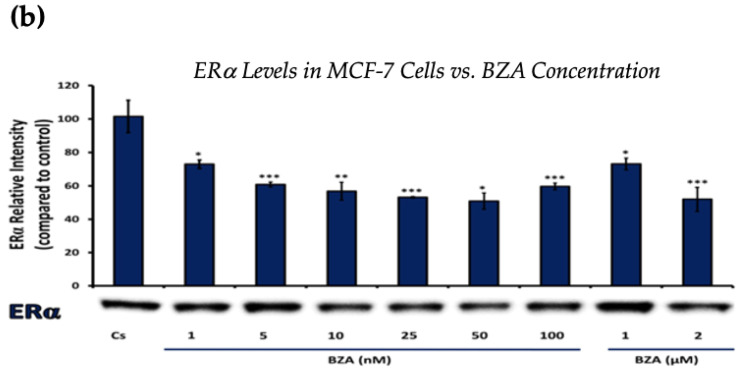
Concentration-dependent effects of BZA on ERα levels in (**a**) T-47D and (**b**) MCF-7 cell lines. The cells were treated for 24 h with varying concentrations of BZA (1 nm–2 μM) and subjected to SDS-PAGE and Western blot analyses, as described in Section 2 (see Figure S1 for additional Western blot images). Protein band densities were quantified using Image Studio Lite. Significance levels were determined based on the following thresholds: * *p* < 0.05, ** *p* < 0.01, and *** *p* < 0.001. Statistical analyses were performed using SPSS (Windows Version 11.5, SPSS Inc.; Chicago, IL, USA).

**Figure 2:**
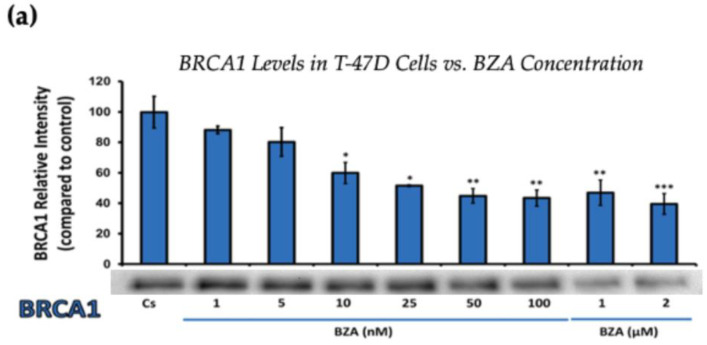

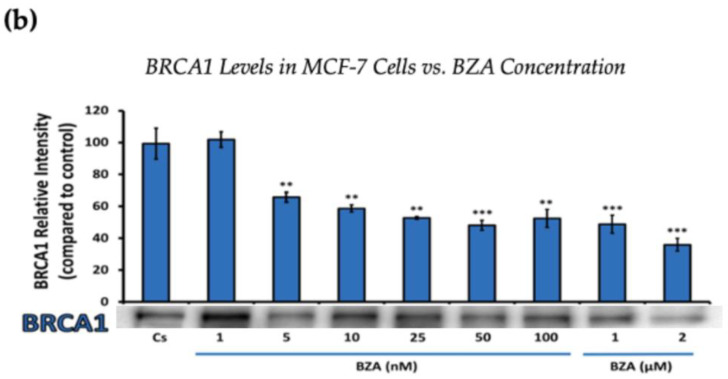
Concentration-dependent effects of BZA on BRCA1 Levels in (**a**) T-47D and (**b**) MCF-7 cell lines. The cells were treated for 24 h with varying concentrations of BZA (1 nM–2 μM) and subjected to SDS-PAGE and Western blot analyses, as described in Section 2 (see Figure S1 for additional Western blot images). Protein band densities were quantified using Image Studio Lite. Significance levels were determined based on the following thresholds: * *p* < 0.05, ** *p* < 0.01, and *** *p* < 0.001. Statistical analyses were performed using SPSS (Windows Version 11.5, SPSS Inc.; Chicago, IL, USA).

Figure 1 and Figure 2 present the results of the concentration dependency studies on the levels of ERα and BRCA1 protein in both T-47D and MCF-7 cell lines. These results were obtained from Western blot analyses. Cells were cultured as previously mentioned and subjected to a 24 h treatment with varying concentrations of BZA, ranging from 1 nM to 2 μM. The findings indicate that there is an increase in BZA concentration correlating with a downregulation of ERα and BRCA1 in both cell lines. Specifically, the treatment of 2 μM BZA resulted in a 63% decrease in ERα levels of T-47D cells, as shown in Figure 1a. A similar trend is evident in MCF-7 cells, with a 48% decrease in the expression of ERα protein when exposed to the 2 μM concentration of BZA, as shown in Figure 1b.

Figure 2 represents the results of BRCA1 levels when T-47D and MCF-7 cells were cultured and treated for 24 h. With increasing BZA treatments, the results demonstrate a decrease in the expression of BRCA1 protein. Notably, the most significant difference is observed with the 2 μM BZA treatment in both cell lines. In Figure 2a, the results from T-47D cells reveal a substantial 61% decrease in BRCA1 expression. Similarly, the results shown in Figure 2b indicate a 64% decrease in BRCA1 levels in MCF-7 cells. The results from the concentration-dependent effects of BZA on both ERα and BRCA1 protein expression denote 2 μM BZA to be the optimal concentration and to further assess ERα and BRCA1 expression in the presence of hormones and antihormones. 

### 3.2. Hormonal and Antihormonal Effects of BZA on ERα and BRCA1 Levels

Establishing the optimal concentration derived from the experimental findings above allowed for further examination of the effects and mechanisms of BZA on cells when treated in different combinations involving E_2_ and ERα antagonists like ICI and TAM. 

Cells underwent a 24 h treatment regimen involving BZA (2 µM), E_2_ (1 nM), and antihormonal agents ICI (1 µM) and TAM (1 µM), administered both individually and in the following combinations: E_2_ + ICI, E_2_ + TAM, E_2_ + BZA, BZA + ICI, and BZA + TAM. Following treatment, cellular proteins were extracted and quantified utilizing the Bradford method, SDS-PAGE, and Western blot analyses.

**Figure 3:**
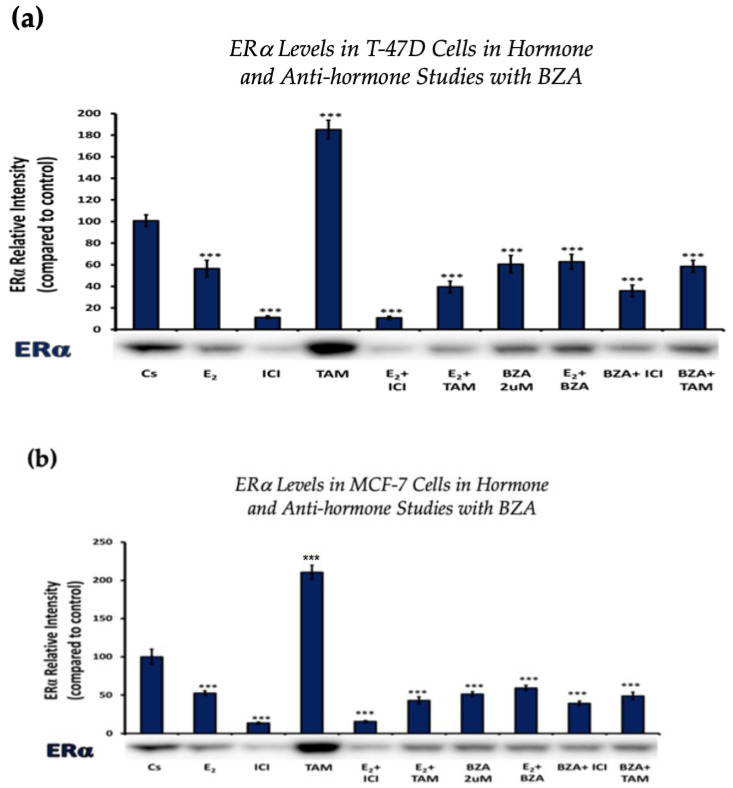
Hormonal and antihormonal effects of BZA on ERα levels in (**a**) T-47D and (**b**) MCF-7 cell lines. The cells were treated for 24 h with varying combinations of BZA (2 μM), hormones, and anti-hormones and subjected to SDS-PAGE and Western blot analyses, as described in Section 2 (see Figure S1 for additional Western blot images). Protein band densities were quantified using Image Studio Lite. Significance levels were determined based on the threshold of *** *p* < 0.001. Statistical analyses were performed using SPSS (Windows Version 11.5, SPSS Inc.; Chicago, IL, USA).

Figure 3 depicts the results of ERα levels resulting from each combination of treatments in T-47D cells and MCF-7 cells. Similar effects are observed when comparing the two cell lines. Treatments involving E_2_ demonstrate a reduction in ERα expression when compared to the control. Similarly, treatments with ICI, a pure ERα antagonist, exhibit a decrease in expression, aligning with expectations. When cells were treated with E_2_ and ICI, there was significant downregulation in comparison to the control. Treatment of BZA, both alone and in combinations, demonstrated a decrease in ERα expression. These effects are consistent in both cell lines with similar results when comparing the various combinations of hormones and antihormones, suggesting that BZA shares similar properties and mechanisms with E_2_ in modulating ERα expression.

**Figure 4:**
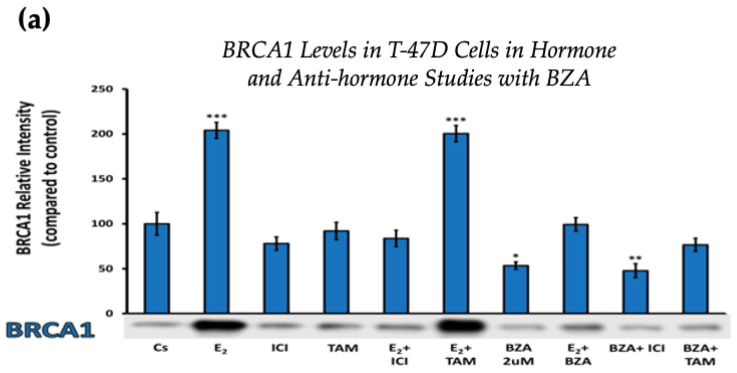

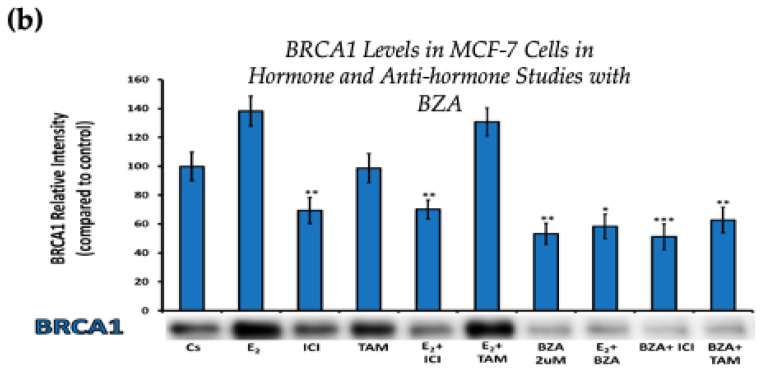
Hormonal and antihormonal effects of BZA on BRCA1 levels in (**a**) T-47D and (**b**) MCF-7 cell lines. The cells were treated for 24 h with varying combinations of BZA (2 μM), hormones, and anti-hormones and subjected to SDS-PAGE and Western blot analyses, as described in Section 2 (see Figure S1 for additional Western blot images). Protein band densities were quantified using Image Studio Lite. Significance levels were determined based on the following thresholds: * *p* < 0.05, ** *p* < 0.01, and *** *p* < 0.001. Statistical analyses were performed using SPSS (Windows Version 11.5, SPSS Inc.; Chicago, IL, USA).

Figure 4 displays the results for BRCA1 levels in response to the various treatment combinations, as previously mentioned. Similar results are seen across both cell lines when compared to the control. Treatment with E_2_ indicates an increased BRCA1 expression as compared to treatment with E_2_ + TAM. However, treatments with ICI, TAM, BZA, or any combination that included BZA reveal decreased BRCA1 protein levels. This suggests that BZA outcompetes E_2_ during co-treatment of BZA + E_2_ with respect to BRCA1 expression.

### 3.3. Effects of BZA on Cell Viability

T-47D and MCF-7 cells (30,000 cells per well) were plated in triplicates into 12-well plates in a medium containing FBS and growth factors for two days. Fresh DCC-FBS medium and ligands were replaced every 48 h. After a seven-day incubation with varying concentrations of BZA (ranging from 1 nM to 2 µM), the cell number was determined using the Cellometer Vision CBA Image Cytometry System. 

**Figure 5:**
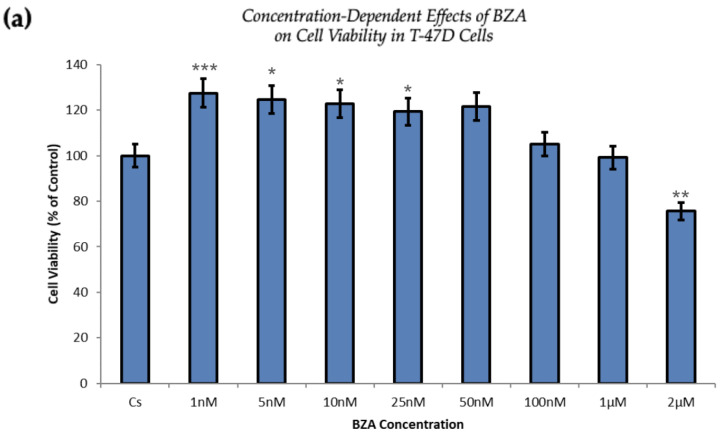

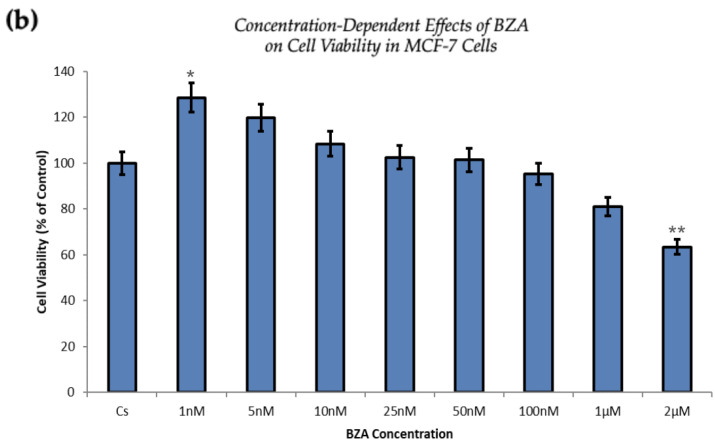
Concentration-dependent effects of BZA on cell viability in (**a**) T-47D and (**b**) MCF-7 cell lines. The cells were treated for 6 days with 1 nM to 2 μM BZA. Cell viability was determined via propidium iodide staining and image cytometry using the Cellometer Vision CBA Image Cytometry System, following the procedures outlined in Section 2. For analysis purposes, the collected samples were compared to the control group. Significance levels were determined based on the following thresholds: * *p* < 0.05, ** *p* < 0.01, and *** *p* < 0.001. Statistical analyses were performed using SPSS (Windows Version 11.5, SPSS Inc.; Chicago, IL, USA).

Figure 5 demonstrates a direct correlation between increasing concentrations of BZA and its capacity to inhibit cell proliferation. At the concentration of 2 µM optimized for experimentation, T-47D cells exhibited a 25% decrease in cell viability, as depicted in Figure 5a. At the same concentration, MCF-7 cells expressed a 37% reduction compared to the control conditions, as displayed in Figure 5b. 

The proliferation of T-47D and MCF-7 cells was increased at lower concentrations of BZA but decreased at higher concentrations, suggesting a dose-dependent drug effect. This implies that the efficacy of BZA increases with higher concentrations. Conversely, the drug may not exhibit a noticeable effect at lower concentrations. This concentration-dependent relationship highlights the importance of dosage considerations when optimizing the therapeutic impacts of BZA.

**Figure 6:**
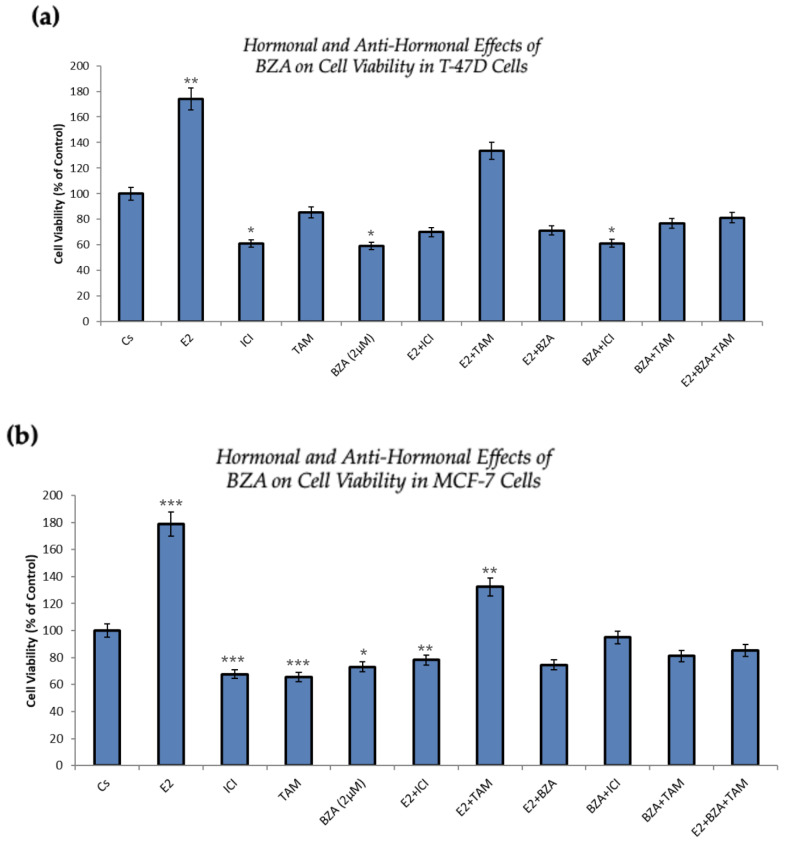
Hormonal and antihormonal effects of BZA on cell viability in (**a**) T-47D and (**b**) MCF-7 cell lines. The cells were treated for 6 days with varying combinations of BZA (2 μM), hormones, and anti-hormones. Cell viability was determined via propidium iodide staining and image cytometry using the Cellometer Vision CBA Image Cytometry System, following the procedures outlined in Section 2. For analysis purposes, the collected samples were compared to the control group. Significance levels were determined based on the following thresholds: * *p* < 0.05, ** *p* < 0.01, and *** *p* < 0.001. Statistical analyses were performed using SPSS (Windows Version 11.5, SPSS Inc.; Chicago, IL, USA).

Figure 6 presents the outcomes of cellular viability assessments under various conditions involving the application of BZA either in isolation or in combination with hormones and antihormones, as previously discussed. In comparison to the control, both cell lines exhibit similar results. As expected, cell numbers increased with the treatment of E_2_, demonstrating the proliferative effects of estradiol on the cells. However, when E_2_ is combined with ICI, antiproliferative effects are observed, in line with ICI’s antiestrogenic properties. When treated with BZA and TAM, both of which are SERMs, alone or in combinations with estrogen or other antihormones, a decrease in cell proliferation is apparent, indicating the selective antiestrogenic properties inherent to SERMs with respect to breast cancer cells. 

### 3.4. Effects of BZA on the Cellular Localization of ERα

**Figure 7:**
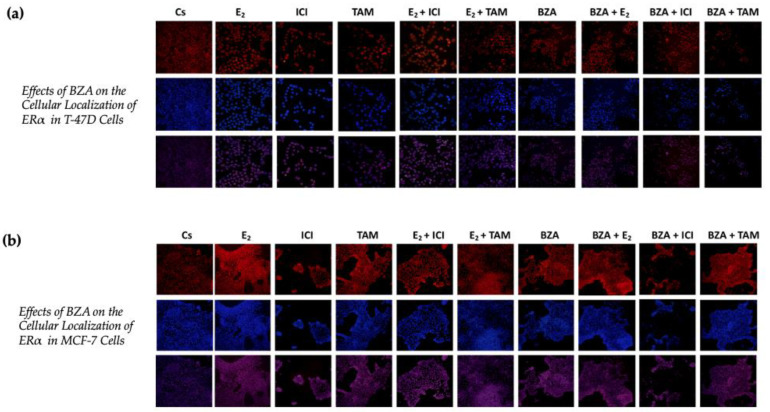
Effects of BZA on the cellular localization of ERα in (**a**) T-47D and (**b**) MCF-7 cell lines. Images were acquired as described in Section 2. The presented results depict the effects on cells exposed to Cy3 and DAPI immunofluorescence stains, corresponding to the row of red-hued and blue-hued images respectively. The row of purple-hued images illustrates the merged overlay of these two staining techniques.

Using confocal microscopy, the cytolocalization of ERα was determined in both cell lines. Figure 7 presents the results of cells when subjected to both Cyanine3 (Cy3; red) and 4′,6-diamidino-2-phenylindole (DAPI; blue) immunofluorescence stains. The results indicate that ERα is located within the nuclei of the cells across all the treatment conditions that were studied in the above experiments. Of significance to this study are the findings associated with the effects of BZA, which indicate a reduction in ERα signal intensity during the treatment conditions. These results align with the consistent patterns observed in the Western blot data, providing further evidence of decreased ERα expression in the experimental context. 

### 3.5. Effects of BZA on T-47D Cells in an Apoptosis Assay

**Figure 8:**
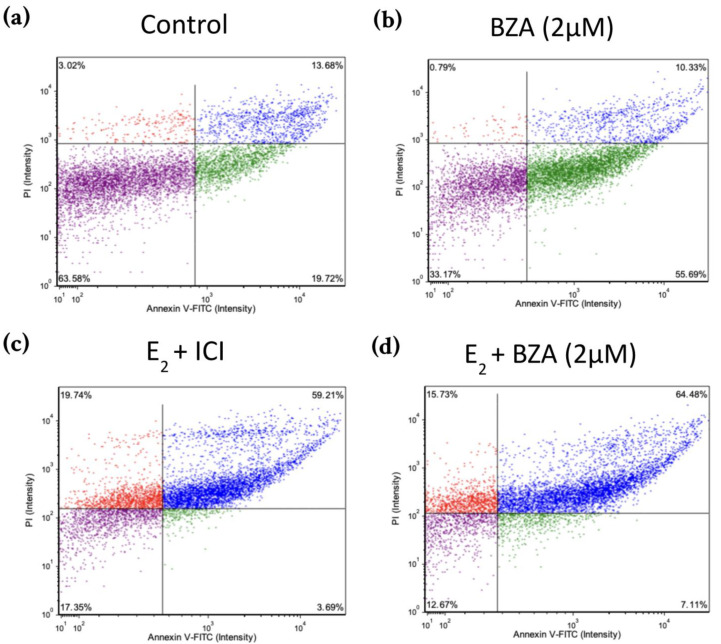

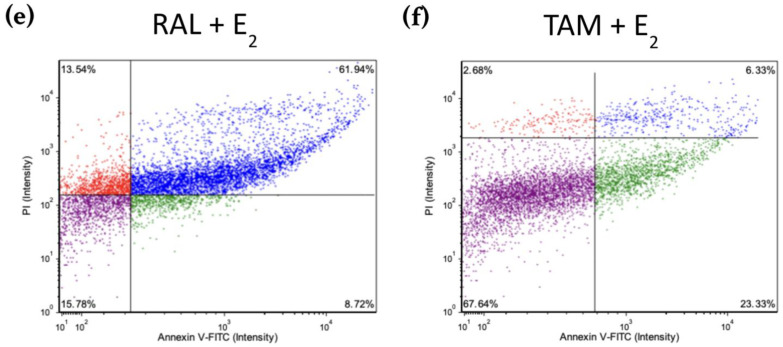
Effects of BZA on T-47D cells in an apoptosis assay. Cells were treated with 2 µM BZA, 1 µM ICI, 1 µM RAL, and 1 µM TAM in a series of combinations for 6 days and then analyzed for Annexin V-PI positivity by image cytometry. Our control treatment was DMSO, which is the solvent used for BZA images that were acquired with the Cellometer Vision CBA Image Cytometry System software. Threshold was set to 0% to measure the total fluorescence of the counted cells. The lower left quadrant (purple) represents live cells; the lower right quadrant (green) represents apoptotic cells; the upper right quadrant (blue) represents necrotic cells; and the upper left quadrant (red) represents debris.

Figure 8 shows scatter plots following treatment with 2 μM BZA and analysis of Annexin V-PI positivity by image cytometry acquired by the Cellometer Vision CBA Image Cytometry System and FCS Express-5 Flow Research Edition. The threshold was set to 0% to measure the total fluorescence of the counted cells. The results reveal that BZA exhibits apoptotic effects on the cells (annexin V-positive only). Necrosis of the cells is observed (annexin V and PI positive) during treatments of E_2_ + ICI, E_2_ + BZA, and E_2_ + RAL. 

### 3.6. Effects of BZA on ESR1 Levels and BRCA1 Levels

**Figure 9:**
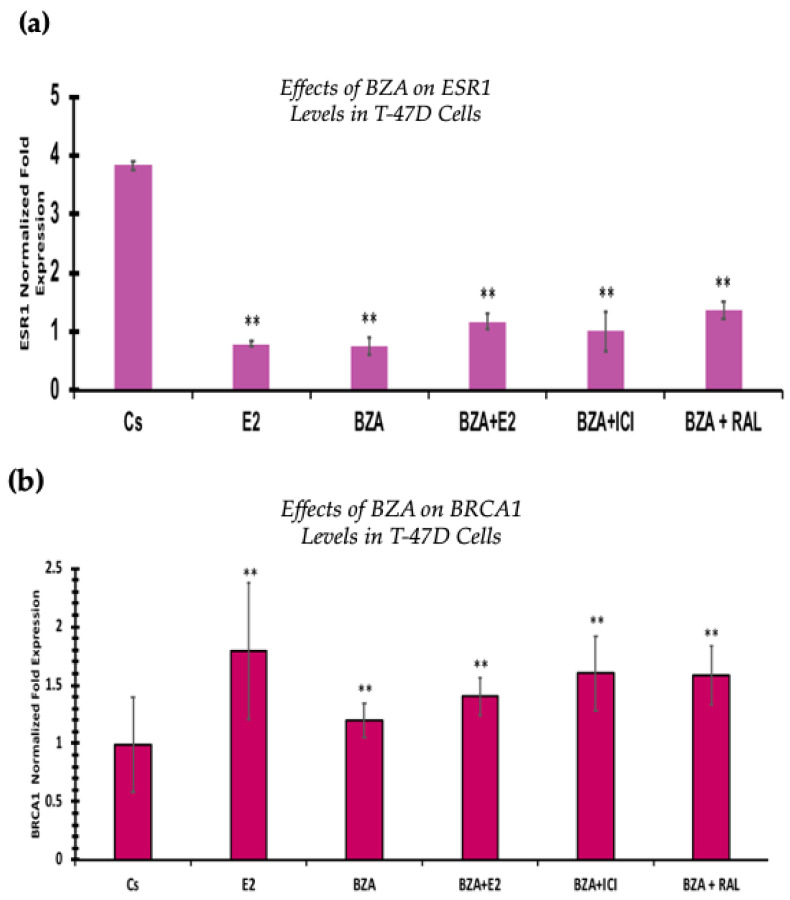
Effects of BZA on (**a**) ESR1 levels and (**b**) BRCA1 levels in the T-47D cell line. ESR1 mRNA levels were determined via RT-qPCR. T-47D cells were treated in the presence or absence of 2 µM BZA, E_2_, ICI, and RAL for 24 h. The results are presented as the mean ± SEM, derived from a minimum of three independent experiments, each comprising three replicates. For analysis purposes, the collected samples were compared to the control group. Significance levels were determined based on the threshold of ** *p* < 0.01 (Kruskal–Wallis test followed by post hoc analysis using Mann–Whitney U-Test).

**Figure 10:**
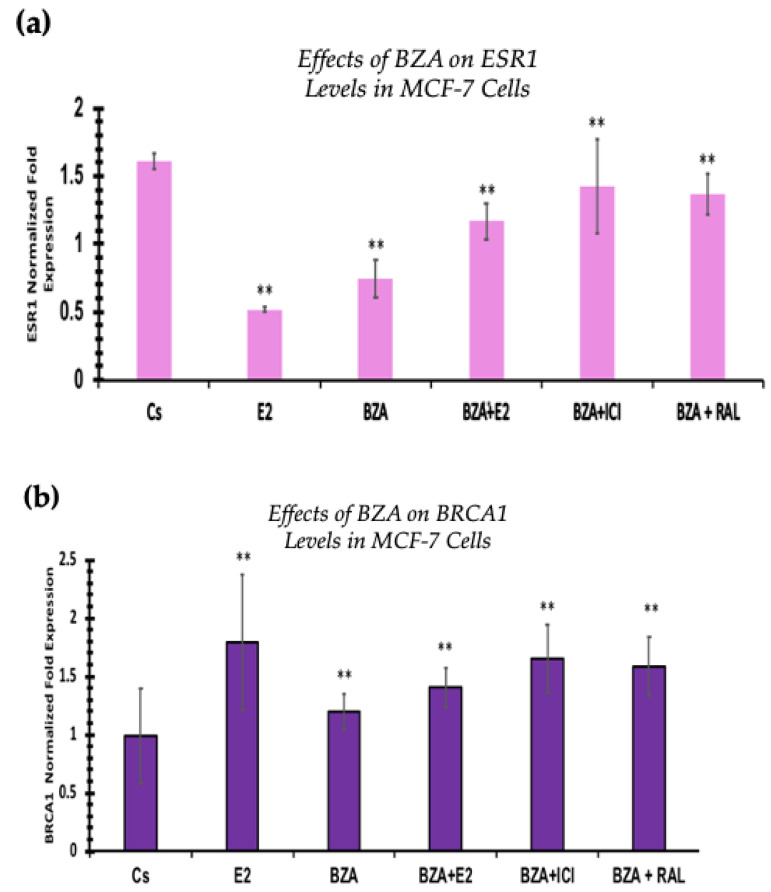
Effects of BZA on (**a**) ESR1 levels and (**b**) BRCA1 levels in the MCF-7 cell line. ESR1 mRNA levels were determined by RT-qPCR. MCF-7 cells were treated in the presence or absence of 2 µM BZA, E_2_, ICI, and RAL for 24 h. The results are presented as the mean ± SEM, derived from a minimum of three independent experiments, each comprising three replicates. For analysis purposes, the collected samples were compared to the control group. Significance levels were determined based on the threshold of ** *p* < 0.01 (Kruskal–Wallis test followed by post hoc analysis using Mann–Whitney U-Test).

RT-qPCR was used to study the outcomes of BZA on ESR1 and BRCA mRNA levels, which are shown in Figure 9 and Figure 10. Cells were treated in the presence or absence of BZA, E_2_, RAL, and ICI for 24 h. Results are shown as the mean ± SEM of at least three independent experiments with three replicates in each experiment. Real-time PCR efficiencies of the target genes (ESR1 and BRCA1) and the reference gene (ACTB) were determined. Results signify that the translational effects discerned in our studies above are supported by the transcriptional results seen during RT-qPCR. Results signify that the translational effects of ER discerned in our studies above are supported by the transcriptional results seen during RT-qPCR. For BRCA1, however, we did not observe similar results with respect to transcriptional and translational expression. 

## 4. Discussion

As previously noted, breast cancer maintains its status as the most frequently diagnosed malignancy worldwide. According to the American Cancer Society’s estimations for 2023, approximately 297,790 new cases of invasive breast cancer will be diagnosed among women in the United States, with an estimated 43,700 women losing their lives to the disease. Overall, the average risk of a woman in the United States developing breast cancer at some point in her lifetime stands at approximately 13% (or a one in eight chance) [33]. This emphasizes the need to study potential therapies for breast cancer treatment.

It was not until the end of the twentieth century that compelling evidence emerged that demonstrated the potential of antiestrogenic agents to delay and possibly prevent the onset of breast cancer in women at risk for the disease [34]. Tamoxifen (TAM), originally developed as an anti-fertility medication by Arthur Walpole, gained significance with the discovery of the estrogen receptor. This discovery enabled Craig Jordan to generate data supporting the notion that TAM could prevent carcinomas [34]. There were concerns centered on the possibility of non-selective antiestrogenic agents inducing adverse effects on bone health or cardiac function. However, clinical investigations into TAM’s efficacy in treating stage IV metastatic breast cancer ultimately allowed for its routine use as an adjuvant endocrine therapy in patients with the earliest stages of ERα-positive tumors [30].

Since then, another medication, Raloxifene (RAL), has been approved to reduce breast cancer risk in postmenopausal women with osteoporosis and postmenopausal women at high risk for the disease [35]. Both drugs belong to the drug class of SERMs. In recent years, Pfizer Inc. has announced FDA approval of DUAVEE™, a conjugated estrogens/BZA drug for the treatment of hot flashes associated with menopause and the prevention of postmenopausal osteoporosis [36]. In addition to TAM, studies are currently in progress to collect data on BZA for its possible approval for use as a routine treatment option for breast cancer in women undergoing menopausal symptoms and at risk of breast cancer [37].

From our studies, Western blot analyses revealed that BZA has concentration-dependent effects on both ERα and BRCA1 protein expression in both T-47D and MCF-7 cell lines. The results of these studies gave rise to the optimal concentration of BZA (2 μM) for the subsequent hormonal studies. In the cellular viability studies, the proliferation of T-47D and MCF-7 cells was increased at lower concentrations of BZA but decreased at higher concentrations, suggesting a dose-dependent drug effect. This implies that the efficacy of BZA increases with higher concentrations. Conversely, the drug may not exhibit a noticeable effect at lower concentrations. This concentration-dependent relationship highlights the importance of dosage considerations when optimizing the potential therapeutic impacts of BZA.

Cell viability assays demonstrated consistent results with hormone study findings, including a dose-dependent decrease in cell proliferation as BZA concentrations increase. In the T-47D cell line, we see a 63% decrease in ERα and a 61% decrease in BRCA1 protein levels. With regard to cell viability, there is a 25% decrease in cell proliferation when compared to the control. As for MCF-7 cells, a similar trend is observed. ERα protein expression decreases by 48% when compared to the control, whereas BRCA1 exhibits a 64% decrease. Following cellular viability, a 37% reduction in MCF-7 cell proliferation is observed when compared to control. This may be suggestive of an interaction between ERα and BRCA1, further supporting the evidence that mutated BRCA1 and ERα signal together and, therefore, contribute to breast tumorigenesis [11].

In the conducted studies, the effects of BZA were compared to other hormones and antihormones. Cellular viability assays demonstrated antiproliferative effects of BZA, with T-47D cell viability decreasing by 40% and MCF-7 cells decreasing by 27% when compared to control. With TAM, similar results were observed, with a 15% decrease in T-47D cell proliferation and a 35% decrease in MCF-7 appreciated. 

Notably, BZA outcompetes E_2_ when a co-treatment of the two is administered. As anticipated, E_2_ on its own exhibits proliferative effects, resulting in a notable increase in proliferation ranging from 74% to 78%. However, with BZA administration and E_2_ coadministration, a reversal is noted, resulting in a 29% reduction in T-47D cells and a 25% reduction in MCF-7 cells. In comparison to TAM, BZA co-treatment with E_2_ demonstrates a significantly greater reduction in cell viability than does TAM and E_2_. 

It is important to note that while TAM is currently utilized in breast cancer treatment, BZA demonstrates a more substantial reduction in cell proliferation when compared to the control than TAM does. This suggests a stronger selectivity toward BZA when in the presence of E_2_. 

Our research studies further illustrate that cells subjected to BZA alone or in conjunction with any of our other treatments displayed a notably elevated rate of cell death through apoptosis compared to necrosis, as depicted in Figure 8. 

In contrast to the previous analyses, which were conducted with TAM, the mRNA analyses were executed utilizing RAL. TAM is beneficial for breast cancer treatment, but its prolonged use presents a significant limitation due to an increased risk of endometrial cancer [23]. RAL, being a newer generation SERM and relatively less extensively studied on the transcriptional level for ESR1 and BRCA1 gene, made it a strategic choice for this investigation to utilize for the mRNA analyses [25]. 

Additionally, the RT-qPCR transcriptional expression of ESR1 levels correlated with the Western blot analyses in both cell lines. However, BRCA1 mRNA levels show similar trends at the translational expression. These effects may be due to other signal transduction factors influencing the expression of BRCA1 mRNA levels. 

When evaluating the effect of BZA on BRCA1 expression, the RT-qPCR results are not consistent with the results of our Western blot analyses in both cell lines. This identifies an alteration at some point in the pathway between transcription and translation of the BRCA1 gene and its associated protein. Moreover, our RT-qPCR results show a significant increase in BRCA1 gene levels after treatment with BZA, while Western blotting demonstrates a significant reduction in BRCA1 protein expression following treatment. These conflicting results are consistent across both cell lines and remain unaffected by the co-treatment of BZA with other hormones and antihormones (E_2_, ICI, RAL). The lack of influence from the hormone co-treatments suggests that the observed discrepancies are likely specific to the action of BZA rather than being mediated through interactions with other hormones. 

One plausible explanation may be attributed to crosstalk mechanisms between steroid receptors and kinase signaling pathways following treatment with BZA. The concept of crosstalk suggests that signals from other pathways can provide alternative regulatory inputs to the BRCA1 gene. In the context of our results, crosstalk mechanisms can provide a pathway for hormone-independent signaling, which may result in alterations of final protein translation. This interplay results in a dynamic interaction that can circumvent the effectiveness of therapies due to modifications that can either resist antagonistic effects or facilitate agonistic responses [38,39]. Additionally, drawing insights from studies on the crosstalk between BRCA1 and Poly [ADP-ribose] polymerase 1 (PARP1), interactions within this crosstalk pathway may contribute to the observed discrepancies. Specifically, the dynamic mechanisms between BRCA1 and PARP1 may be suggestive of a complex regulatory network that can influence gene expression and protein translation [40]. 

In addition, studies have demonstrated RNA-induced BRCA1 gene methylation, which reduced BRCA1 protein expression [41]. We cannot rule out the possibility of BZA influencing crosstalk or gene methylation. However, this is outside the scope of our current discussion. Additional studies are necessary to determine the specific mechanism by which BZA induces alterations in the transcription and translation of the BRCA1 gene and its associated protein.

Moreover, confocal imaging revealed that cytolocalization of ERα remained unaltered upon treatment with BZA.

## 5. Conclusions

The findings of our research contribute to understanding the effects of BZA on breast cancer cells. It is evident that BZA demonstrates antiestrogenic effects on breast cancer cells, akin to SERMs, highlighting its potential as a promising antiestrogenic agent in breast cancer therapy. However, further research is warranted to validate its clinical use and elucidate its precise mechanism of action, which may provide valuable insights into its potential for FDA approval as a breast cancer treatment option.

## Data Availability

The data that support the findings of this research project are available upon request from the corresponding authors (M.S. and S.D.).

## Supplementary Materials

The following supporting information can be downloaded at: https://www.mdpi.com/article/10.3390/cancers16040699/s1, Figure S1: additional Western blot images.
