# Supplementary material for: Modulation of Estrogen Receptor Alpha (ERα) and Tumor Suppressor Gene BRCA1 in Breast Cancer Cells by Bazedoxifene Acetate (BZA)
Source: Cancers (Basel). 2024 Feb 7;16(4):699. doi: 10.3390/cancers16040699 (PMC10886716; doi:10.3390/cancers16040699)
Supplement: Supplementary file 1 [file cancers-16-00699-s001.zip › cancers-2701746-supplementary.pdf]

cancers

MDPI

Supplementary Materials: Modulation of Estrogen Receptor Alpha (ERα) and Tumor Suppressor Gene BRCA1 in Breast Cancer Cells by Bazedoxifene Acetate (BZA)

Monica Szmyd, Aisha Zanib, Victoria Behlow, Erin Hallman, Samantha Pfiffner, Raquel Yaldo, Nina Prudhomme, Katelyn Farrar, and Sumi Dinda

T-47D ERα
Concentration Dependency

Band	Average Densitometry
CS	1.01774616
1nM	0.79112085
5nM	0.58932657
10nM	0.55326012
25nM	0.4668756
50nM	0.40082506
100nM	0.44453847
1µM	0.5235239
2µM	0.37234814

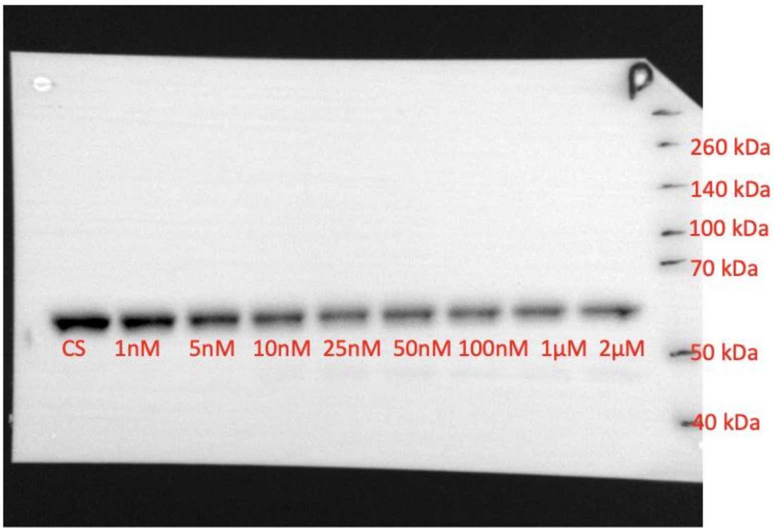

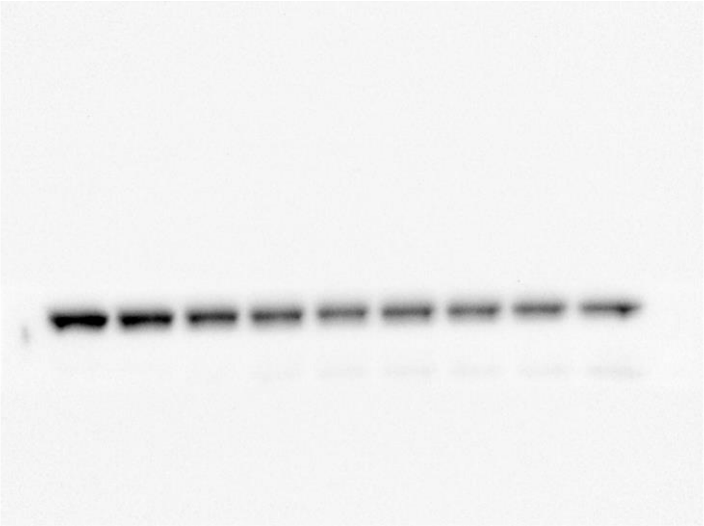

a) Additional Western blot images for Figure 1a

MCF-7 ERα
Concentration Dependency

Band	Average Densitometry
CS	1.01574242
1nM	0.67757433
5nM	0.59059821
10nM	0.51440963
25nM	0.53153743
50nM	0.50827844
100nM	0.66100829
1μM	0.69804639
2μM	0.90448398

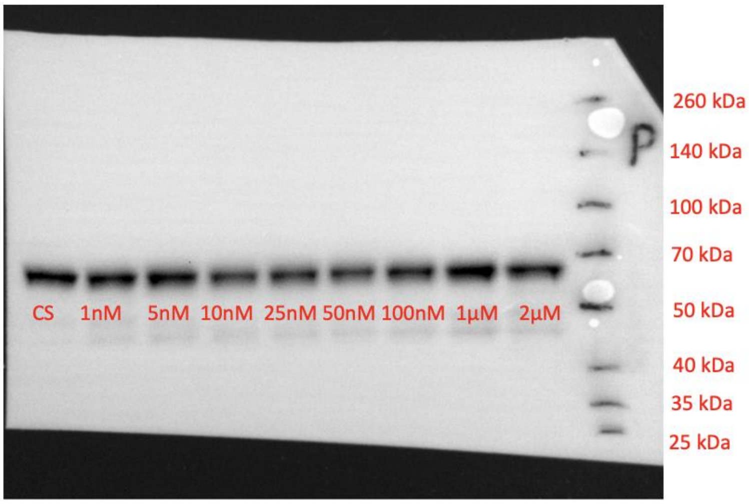

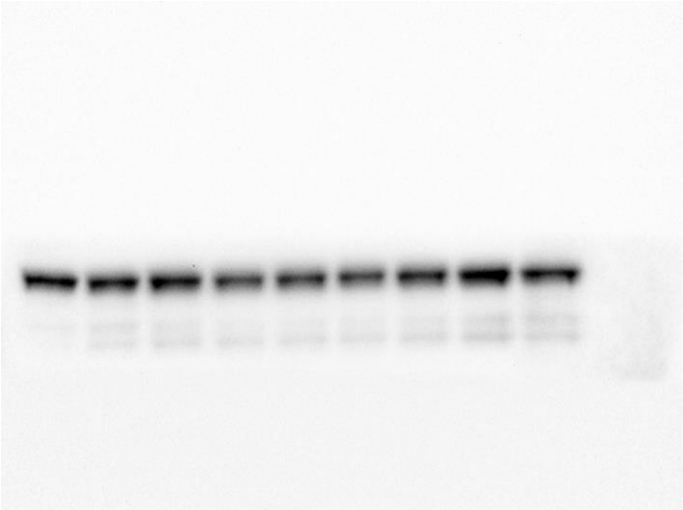

b) Additional Western blot images for Figure 1b

T-47D BRCA1
Concentration Dependency

Band	Average Densitometry
CS	0.99851278
1nM	0.88168429
5nM	0.80220687
10nM	0.44890996
25nM	0.5154895
50nM	0.49707698
100nM	0.49894638
1µM	0.4695538
2µM	0.39503911

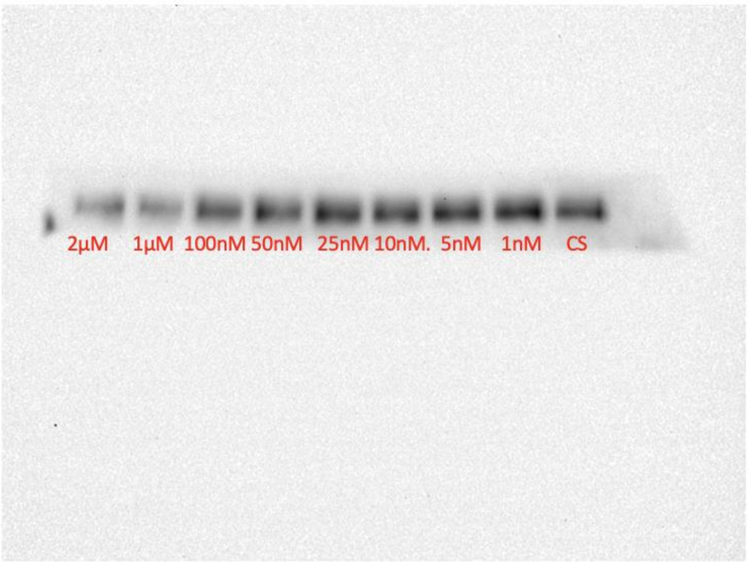

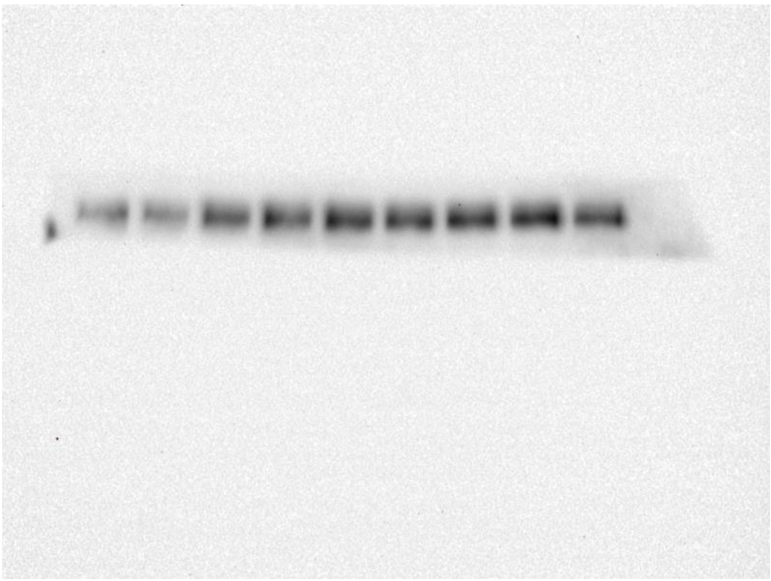

c) Additional Western blot images for Figure 2a

MCF-7 BRCA1
Concentration Dependency

Band	Average Densitometry
CS	0.99280734
1nM	0.99529657
5nM	0.63315416
10nM	0.58665259
25nM	0.52784811
50nM	0.41943118
100nM	0.61543179
1µM	0.60349353
2µM	0.35996514

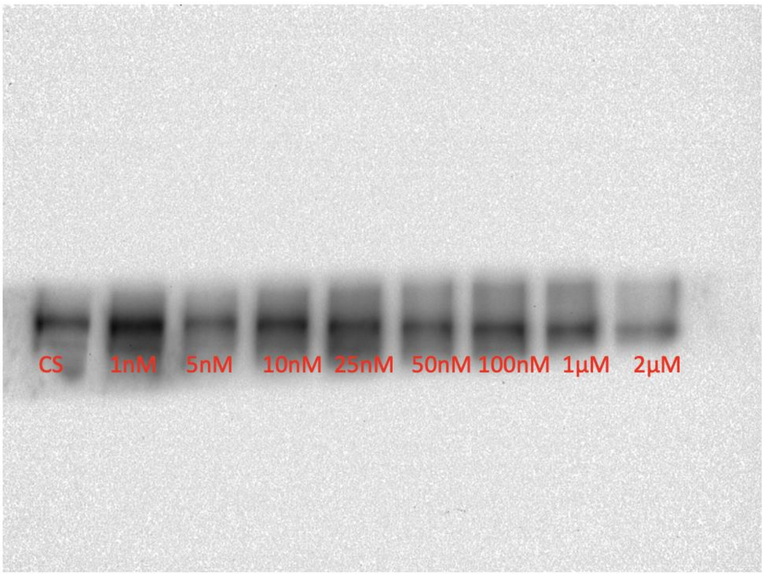

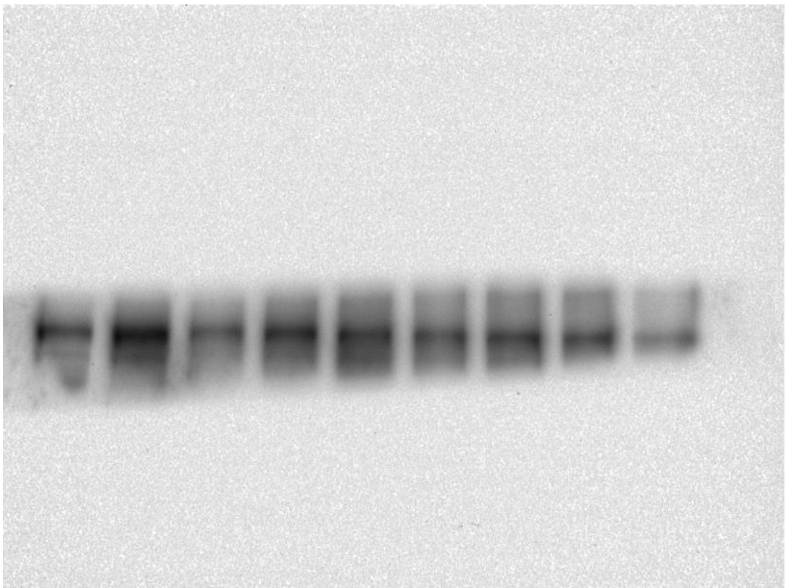

d) Additional Western blot images for Figure 2b

T47D ERα
Hormone Study

Band	Average Densitometry
CS	1.00811679
E2	0.56445809
ICI	0.11401044
TAM	1.85192589
E2 + ICI	0.10875479
E2 + TAM	0.39514937
BZA (2μM)	0.60462987
E2 + BZA (2μM)	0.62776008
BZA (2μM) + ICI	0.35881244
BZA(2μM) + TAM	0.58441278

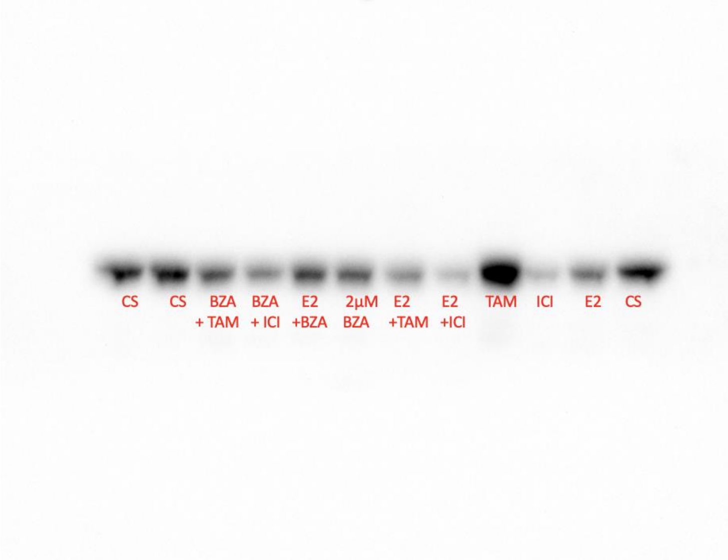

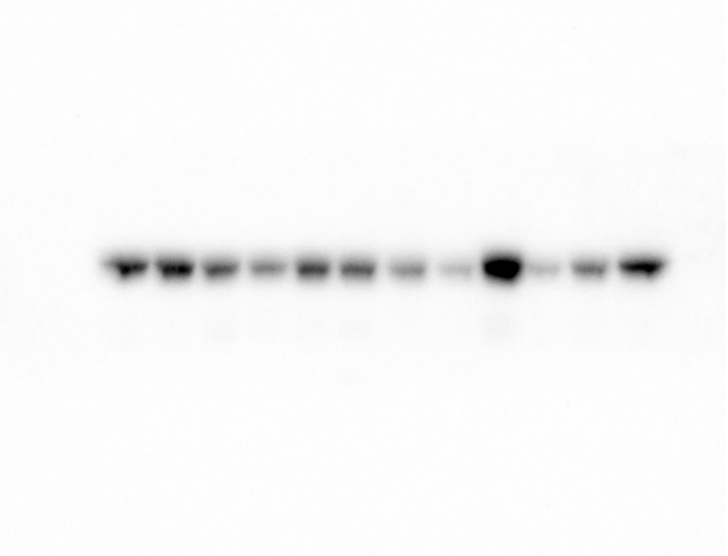

e) Additional Western blot images for Figure 3a

MCF-7 ERα
Hormone Study

Band	Average Densitometry
CS	1
E2	0.52436688
ICI	0.13683602
TAM	2.10427627
E2 + ICI	0.15754149
E2 + TAM	0.42972385
BZA (2µM)	0.51192426
E2 + BZA (2µM)	0.59276325
BZA (2µM) + ICI	0.39208286
BZA(2µM) + TAM	0.48722512

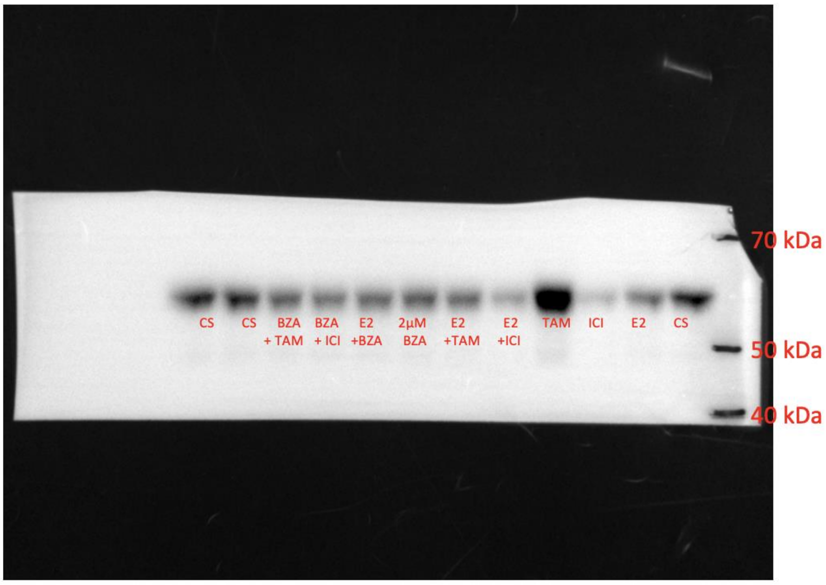

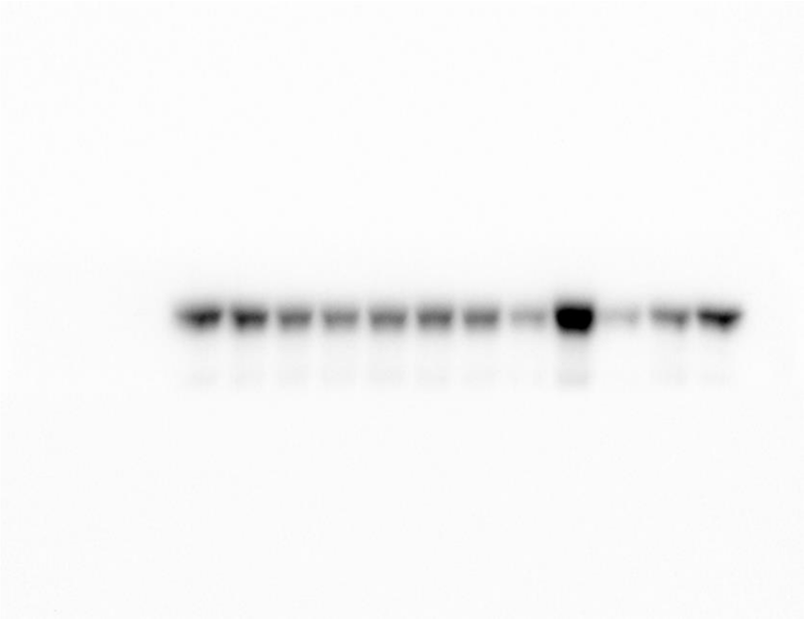

f) Additional Western blot images for Figure 3b

T-47D BRCA1
Hormone Study

Band	Average Densitometry
CS	1
E2	2.03992807
ICI	0.78077046
TAM	0.92018429
E2 + ICI	0.83644473
E2 + TAM	2.00376388
BZA (2µM)	0.534663
E2 + BZA (2µM)	0.99213444
BZA (2µM) + ICI	0.47896857
BZA(2µM) + TAM	0.76663255

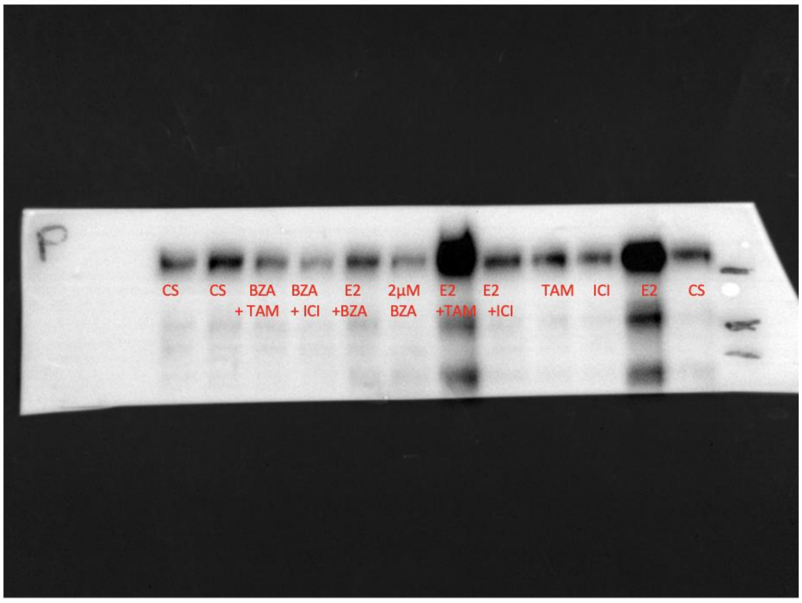

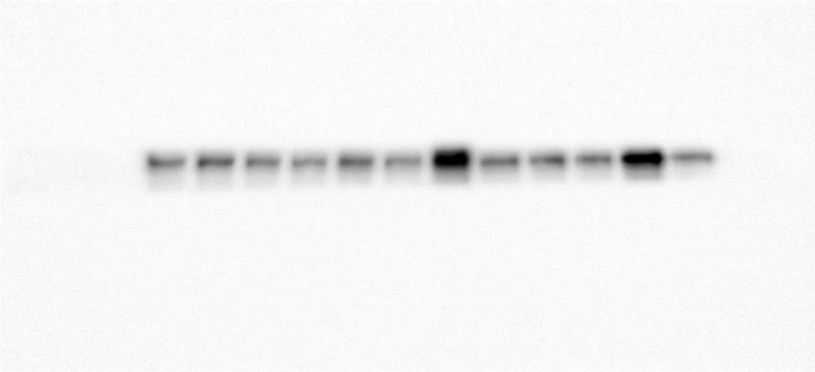

g) Additional Western blot images for Figure 4a

MFC-7 BRCA1
Hormone Study

Band	Average Densitometry
CS	0.99850118
E2	1.38260028
ICI	0.69392258
TAM	0.98790735
E2 + ICI	0.70157159
E2 + TAM	1.30778452
BZA (2µM)	0.53236416
E2 + BZA (2µM)	0.58331407
BZA (2µM) + ICI	0.51091619
BZA(2µM) + TAM	0.62804048

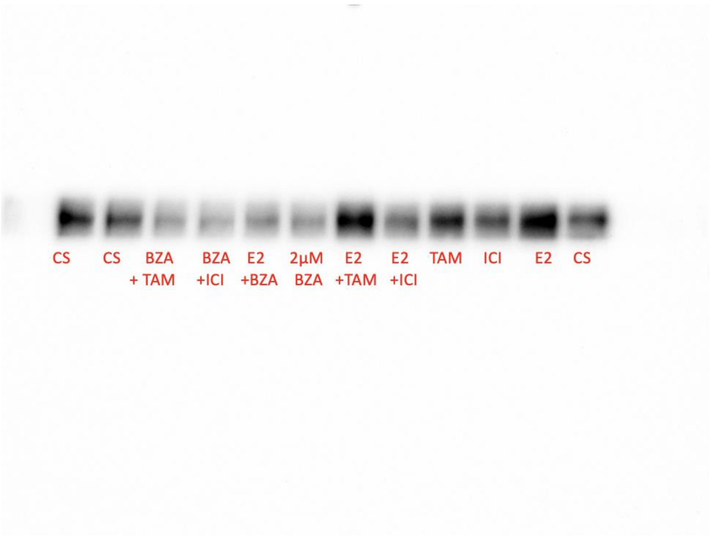

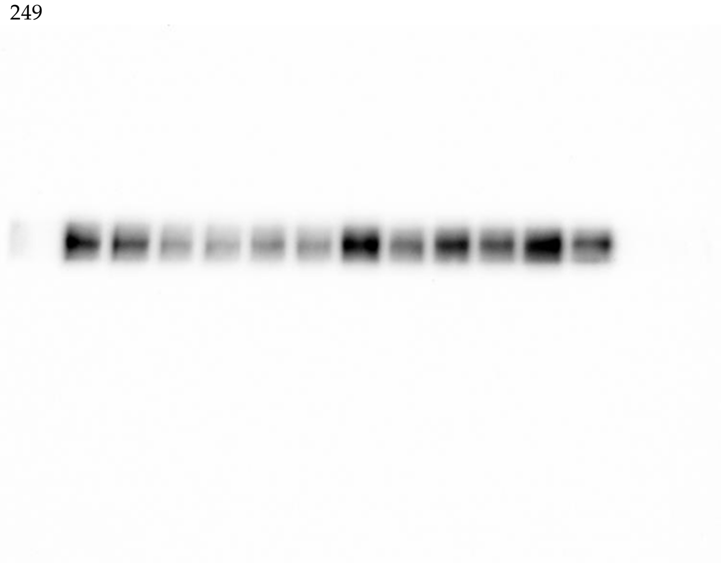

h) Additional Western blot images for Figure 4b

Figure S1. For each Western blot figure in the paper, we have provided additional images and average densitometry data. Please note that the gel images presented in our publication were condensed to fit our graph formatting, and PDVF membranes were cut and may appear to be cut in certain cases in order probe BRCA1 and ERα from a single membrane.
